# Editorial Notes

**Published:** 1931

**Authors:** 


### Editorial Notes

**The Queen Victoria Jubilee Convalescent Home.**

It is difficult for some of us to realize that thirty-four years have passed since Queen Victoria's Diamond Jubilee; yet the fact that since its opening the Convalescent Home has treated nearly 50,000 patients reminds us of the long and valuable service that this institution has rendered. Although its work has been largely that of relief to the two great general hospitals—whence it has been able since 1920 to receive a certain number of cot cases—its usefulness is by no means limited to this. Many patients come direct from their own homes—a godsend to the man or woman trying to recover from an acute illness in the unhelpful surroundings of a cramped and overcrowded house. We venture to commend this possibility to the consideration of the general practitioners of Bristol and its neighbourhood, adding that these benefits are usually to be had for nothing, except in the case of a few patients who need to stay longer than the customary fortnight. From these a fee of a guinea for each extra week is required. On application to the Matron the requisite forms of admission will be forthcoming. Of these there are two; one, a simple guarantee of character to be signed by some responsible person; the other, a form of medical fitness (which is probably familiar to most Bristol doctors), excluding tuberculous, cardiac and infectious disease, to be signed by the practitioner in attendance.

Although persons are expected to be convalescent, and therefore not in need of active or elaborate treatment, they are safeguarded against the risk of unperceived relapse by the care of the nursing staff and by the daily visit of one or other of the medical officers. We repeat that for persons of straitened means recovering from acute illness or injury the quiet, orderly, open-air life of the Convalescent Home will often prove ideal.

**The "Lancet" Commission on Nursing.**

IN December last the *Lancet* appointed a Commission to inquire into the reasons for the shortage of candidates for the profession of sick nursing, and to offer suggestions for making the service more attractive to suitable women. Two interim reports have now been published, of which the second appeared in the *Lancet* for 15th August. The questionnaire was sent out to 1,031 hospitals, of which 686 (or 67 per cent.) sent replies. From certain classes of hospitals the percentage of replies was low, notably tuberculosis hospitals and some groups of hospitals not wholly approved for complete training. It may not be unfair to guess that the conditions of service in the non-replying hospitals were not above the average. From the variety of explanations advanced by the replying hospitals it is evident that their managers are entirely ignorant of the true reasons for the shortage of nurses, and we venture to doubt whether of their own initiative our hospital managers are likely to be able to attract suitable women in large enough numbers to satisfy the demand for trained or even untrained sick nurses. A clear summary of the evidence is given in this second interim report. The summary is presented without critical comment, and is a singularly fair account of what is demanded of the nurse herself and what is offered in return. The impression we have gained from reading the whole report is that a modern nurse is required to be capable of receiving an academic education little if at all lower than the pass standard for an ordinary B.A. degree as well as being trainable in a highly skilled handicraft, which is not an additional subject in any "pass" B.A. examination. Finally, she must possess an inexhaustible fund of patience and sympathy such as is not expected of doctors or even of ministers of religion themselves. We shall be interested to see how the *Lancet* proposes that hospitals shall obtain the nursing staffs they need.

